# Neuronal Conduction of Excitation without Action Potentials Based on Ceramide Production

**DOI:** 10.1371/journal.pone.0000612

**Published:** 2007-07-18

**Authors:** Caroline Fasano, François Tercé, Jean-Pierre Niel, Hang Thi Thu Nguyen, Abel Hiol, Justine Bertrand-Michel, Nicole Mallet, Xavier Collet, Jean-Pierre Miolan

**Affiliations:** 1 Laboratoire de Physiologie Neurovégétative, UMR CNRS 6153-INRA 1147, Institut Fédératif de Recherche Jean Roche IFR 11, Université Paul Cézanne, Aix-Marseille III, Faculté des Sciences et Techniques, Marseille, France; 2 Plateau Technique de Lipidomique, INSERM IFR 30/Toulouse Génopole, INSERM U563, Hôpital Purpan, Toulouse, France; 3 Laboratoire de Chimie Biologique Appliquée, UMR-INRA 1111, Université Paul Cézanne, Aix-Marseille III, Faculté des Sciences et Techniques, Marseille, France; 4 INSERM U563, Département Lipoprotéines et Médiateurs Lipidiques, IFR 30, CPTP, Hôpital Purpan, Toulouse, France; Freie Universitaet Berlin, Germany

## Abstract

**Background:**

Action potentials are the classic mechanism by which neurons convey a state of excitation throughout their length, leading, after synaptic transmission, to the activation of other neurons and consequently to network functioning. Using an in vitro integrated model, we found previously that peripheral networks in the autonomic nervous system can organise an unconventional regulatory reflex of the digestive tract motility without action potentials.

**Methodology/Principal Findings:**

In this report, we used combined neuropharmacological and biochemical approaches to elucidate some steps of the mechanism that conveys excitation along the nerves fibres without action potentials. This mechanism requires the production of ceramide in membrane lipid rafts, which triggers in the cytoplasm an increase in intracellular calcium concentration, followed by activation of a neuronal nitric oxide synthase leading to local production of nitric oxide, and then to guanosine cyclic monophosphate. This sequence of second messengers is activated in cascade from rafts to rafts to ensure conduction of the excitation along the nerve fibres.

**Conclusions/Significance:**

Our results indicate that second messengers are involved in neuronal conduction of excitation without action potentials. This mechanism represents the first evidence—to our knowledge—that excitation is carried along nerves independently of electrical signals. This unexpected ceramide-based conduction of excitation without action potentials along the autonomic nerve fibres opens up new prospects in our understanding of neuronal functioning.

## Introduction

The prevertebral ganglia (coeliac, superior mesenteric, inferior mesenteric and major pelvic ganglia) are anatomical relays, located in the abdominal cavity, along the autonomic sympathetic fibres innervating the viscera. However, they are physiologically able to regulate autonomic functions such as digestive tract motility, vascular motility, secretion and absorption [1]–[5]. Indeed, they are established peripheral nervous centres with many well-recognized integrative properties such as convergence of central inputs, projections of the visceral inputs at the pre- and post-synaptic level, gating by the central fibres of the projection of the visceral inputs, pacemaker activity [5]. These ganglia represent valuable models for the study of neuronal functioning which explains why they have been studied for more than 30 years using electrophysiological, pharmacological, immunohistochemical and biochemical techniques.

We found previously that peripheral networks connected to the coeliac plexus can organize an unconventional regulatory reflex of the digestive tract motility, the gastroduodenal inhibitory reflex (GIR). This reflex leads to inhibition of the duodenal motility in response to distension of the stomach and is very likely involved in gastric emptying. The afferent and efferent limbs of this reflex are represented by gastric mechanosensitive afferent fibres projecting on the coeliac plexus and by ganglionic neurons innervating the duodenum, respectively since the reflex is abolished after section of the nerve trunks connecting the coeliac plexus to the viscera [1]. We first provided pharmacological and electrophysiological evidence that this reflex is organized without action potentials along the autonomic nerve fibres by an unconventional mechanism of excitation which remains unexplained [1]. We then provided pharmacological evidence that during GIR, the neurotransmitter released by the afferent fibres to activate the ganglion neurons is nitric oxide (NO) [2].

It now remains to analyse the molecular mechanisms involved in the conduction of excitation without action potentials along autonomic nerve fibres during GIR. For that purpose, we have used an *in vitro* preparation consisting of the coeliac plexus connected to the stomach and duodenum (Figure S1). We hypothesized that molecules other than ions might be involved in the conveyance of excitation along the nerve fibres. This suggested that second messengers could play a role in this mechanism of neuronal communication. Among them, the sphingolipids represent an overlooked pool of molecules. One of their metabolites, ceramide, is produced within the neuronal membrane but activates many second messengers thus playing a central role in the signalling of important physiological events such as cell differentiation, growth, apoptosis and calcium homeostasis [6]–[8]. We have therefore looked for the involvement of ceramide in the mechanism of conduction of excitation without action potentials.

## Results

### Gastroduodenal inhibitory reflex

**Figure 1:**
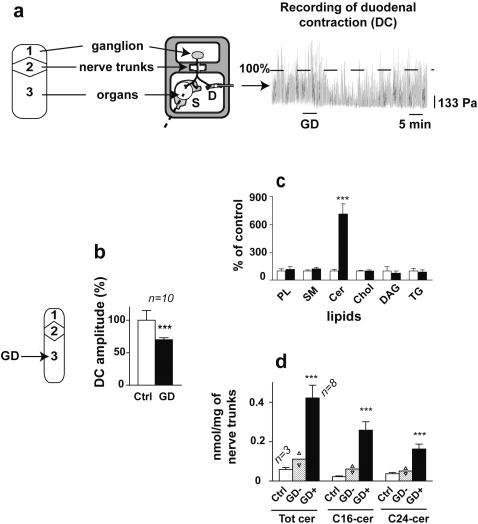
Gastric distension triggers gastroduodenal inhibitory reflex and concomitant production of ceramide in the nerve fibres. a, Schematic representation of the organ bath (S, stomach; D, duodenum; GD, gastric distension: see Materials and Methods) and manometric recordings of duodenal contractions before and during GIR. GD triggered a decrease in the amplitude of the duodenal contractions characterising the GIR. The dashed horizontal line is the control indicating the mean amplitude of the duodenal contractions over the period preceding the distension shown in the recordings. b, histogram of the mean amplitude of the duodenal contractions expressed as % of control±SEM. DC, duodenal contraction; Ctrl, control; GD, GIR after gastric distension. c, lipid content of the nerve trunks before and during GIR (see Materials and Methods). PL, phospholipids; SM, sphingomyelin; Cer, ceramide; chol, cholesterol; DAG, diacylglycerols; TG, triglycerides. White bars, control, n = 3; black bars, during GIR, n = 8. Results are given as the percentage of control±SEM. d, quantification of ceramide production during GIR expressed in nM/mg of nerve trunks. Ctrl, control; GD-, after gastric distension not triggering GIR; GD+ , after gastric distension triggering GIR; *** results are significant in a Student's t test with p<0.001.

Under our experimental conditions the duodenum showed spontaneous phasic contractions with a frequency of 10–20 min^−1^ and an amplitude of 250–650 Pa. Following gastric distension (2500 Pa for 5 min) the mean amplitude of duodenal contractions was 68±15% of control which revealed a significant inhibition of duodenal contractions (paired t test, P<0.001, df = 9). This phenomenon had a mean latency of 5±2 min and a mean duration of 17±4 min: it characterized the GIR organized by the coeliac plexus (Fig. 1a, b). In previous studies, we demonstrated that this reflex is unaffected by selective superfusion of the coeliac plexus with tetrodotoxin (a sodium channel blocker) 3.1 µM for 10–20 min. Under these experimental conditions, all action potentials elicited in the coeliac neurons by peripheral nerve stimulation or by direct stimulation with the microelectrode were abolished. GIR was also unaffected by selective superfusion of the peripheral nerves with a sodium free solution for at least 15 min. Under these conditions, antidromic and synaptic action potentials elicited by peripheral nerve stimulation were abolished, while the neurones remained able to fire action potentials when directly stimulated by the microelectrode. On the basis of these results, the involvement of sodium action potentials in the organization of the GIR could be ruled out [1]. We also showed that GIR was unaffected when the peripheral nerves were selectively superfused for at least 15 min with cadmium chloride 10^3^ µM (an inorganic calcium channel blocker), or calcium free solution. Under these conditions, antidromically, synaptically and directly-induced action potentials were still elicited in the coeliac neurons. This ruled out the involvement of calcium action potentials in the organization of the GIR [1]. Taken together, all these results show that sodium or calcium action potentials are not involved in the organization of the GIR. As these action potentials are the only ones fired by mammalian neurons, we concluded that GIR was organized without action potentials along the afferent and efferent neurons. As the prevertebral neurons and the visceral afferents fire sodium action potentials, we concluded that these neurons have two levels of activity, one involving the genesis of action potentials and probably involved in fast regulation and the other without action potentials and probably involved in slow regulation [4].

### Recurrent production of ceramide along the nerve fibres during the GIR

**Figure 2:**
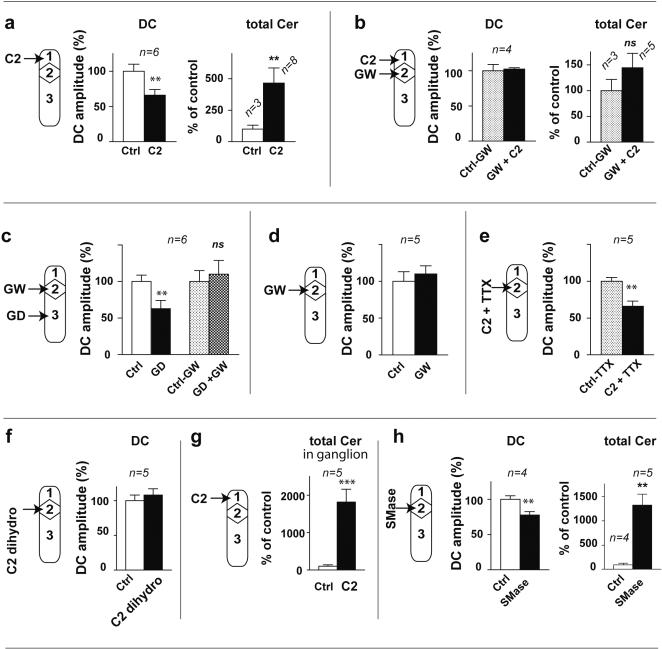
Recurrent production of ceramide during the organization of the GIR. Recording of duodenal contraction (DC) and determination of ceramide content (Cer) on the nerve trunks were performed as in Fig. 1 following different treatments. Results are expressed as the percentage of control±SEM for each series of experiments. a, superfusion of the ganglion compartment with 6 µM C2-ceramide for 5 min. Ctrl, control; C2, C2-ceramide. b, superfusion of the nerve trunks compartment with 16 µM GW4869 for 30 min, then of the ganglion with 6 µM C2-ceramide. Ctrl-GW, control in the presence of GW; GW+C2, C2-ceramide in the presence of GW. c, GD performed before and after superfusion of the nerve trunks with 16 µM GW4869 for 30 min. Ctrl, control; GD, gastric distension; Ctrl-GW, control in the presence of GW; GD+GW , gastric distension in the presence of GW. d, recording of duodenal contractions before and after superfusion of the nerve trunks with 16 µM GW4869 for 30 min.Ctrl, control; GW, superfusion of the nerve trunks with GW. e, superfusion of the nerve trunks with 6 µM C_2_-ceramide in the presence of 3 µM tetrodotoxin. Ctrl-TTX, control in the presence of TTX; C2+TTX, C2-ceramide in the presence of TTX. f, superfusion of the nerve trunks with 6 µM C_2_-dihydroceramide. Ctrl, control; C2-dihydro, superfusion of the nerve trunks with C2-dihydroceramide. g, superfusion of the ganglion compartment with 6 µM C2-ceramide for 5 min. Ctrl, control; C2, superfusion of the coeliac plexus with C2-ceramide, . h, superfusion of the nerve trunks with sphingomyelinase (1 UI/100 µl). Ctrl, control; SMase, superfusion of the nerve trunks with SMase . Differences with control were significant in a Student's t test, *** p<0.001; ** p<0.01; * p<0.05 or non significant (ns).

To investigate whether lipid second messengers were involved in the conduction of excitation without action potentials, we analyzed the lipid composition of the nerve trunks connecting the coeliac plexus to the viscera following gastric distension. We clearly observed that during the GIR, among all major lipid classes only ceramide selectively increased 7-fold (unpaired t test, P<0.001, df = 9, Fig. 1c). This production is 0.42±0.07 nM/mg of nerve trunks (unpaired t test, p<0.001, df = 9, Fig. 1d). Interestingly, ceramide production involved mainly long-chain (12.6-fold increase in C_16_ species) and to a lesser extent very long-chain ceramides (3 and 5.7-fold increase for C_24∶0_ and C_24∶1_ species, respectively, Fig. 1d). In addition, in two experiments where gastric distension did not trigger the GIR, the ceramide content only increased less than 2-fold (Fig. 1d). These results suggest that ceramide production in the nerve fibres is necessary for the organisation of GIR. To confirm this assumption, we treated the preparation with different compounds known to interfere with the ceramide pathway. As shown in Fig. 2a, in the absence of gastric distension, after superfusion of the coeliac plexus with 6 µM C_2_-ceramide, a permeant analogue of ceramide [9], [10], the mean amplitude of duodenal contractions was 66±8% of control which revealed a significant inhibition of duodenal contractions (paired t test, P<0.01, df = 5). This phenomenon had a mean latency of 8±1 min and a mean duration of 15±3 min, thus mimicking the GIR organized by the coeliac plexus. In one additional experiment, we checked that section of the nerve trunks connecting the coeliac plexus to the viscera abolished the inhibition of duodenal contractions following superfusion of the coeliac plexus with 6 µM C_2_-ceramide. These data allow us to exclude any paracrine signalling in the propagation of the excitation. It confirms the results of a previous study where we showed that GIR was blocked after section of the nerve trunks connecting the coeliac plexus to the viscera [1]. Concomitantly to the inhibition of the duodenal contractions, superfusion of the coeliac plexus with 6 µM C_2_-ceramide induced an increase in the ceramide level in the nerve trunks 4.7 fold (unpaired t test, P<0.01, df = 9, Fig. 2a). This increase is similar to that obtained following gastric distension and involves C16 as well as C24 molecular species. Moreover, both the inhibition of the duodenal contractions and the ceramide production were abolished when the nerve trunks were superfused for at least 30 min with 16 µM GW4869, a selective inhibitor of neutral sphingomyelinase [11], [12] before superfusion of the coeliac plexus with C_2_-ceramide (Fig. 2b). Therefore a local increase in the concentration of ceramide along the nerve fibres fails to affect duodenal motility if ceramide production downstream is blocked. We confirmed the involvement of ceramide in the organisation of the reflex by showing that superfusion of the nerve trunks with GW4869 blocked the GIR triggered by gastric distension (Fig. 2c). However, in the absence of gastric distension, superfusion of the nerve trunks with GW4869 did not significantly affect the amplitude of duodenal contractions (paired t test, non significant, df = 4, Fig. 2d). This result indicates that under our experimental conditions, the basal sphingomyelinase activity and the ceramide production were too low to produce an inhibition of duodenal motility. When C_2_-ceramide was superfused on the nerve trunks in the presence of 3 µM tetrodotoxin, the mean amplitude of duodenal contractions was 66±7% of control which revealed a significant inhibition of duodenal contractions (paired t test, P<0.01, df = 4, Fig. 2e) indicating that the effect of C_2_-ceramide is independent of action potentials. Superfusion of the nerve trunks with 6 µM C_2_-dihydroceramide, an inactive analogue of ceramide [9], [10], did not affect duodenal contractions, indicating that the effect of C_2_-ceramide was specific (paired t test, non significant, df = 4, Fig. 2f). Nowadays it is acknowledged that permeant short-chain ceramides mimic the effect of natural ceramide [7], [13]–[16]. C_2_-ceramide can in fact be converted to long chain ceramide [15], [16], a process that we observed in the ganglion treated with C_2_-ceramide (18-fold increase in total ceramide, unpaired t test, P<0.01, df = 10, Fig. 2g). C_2_-ceramide may also activate a neutral sphingomyelinase which in turn generates endogenous ceramide within minutes [17], a process compatible with the latency of the duodenal inhibition triggered by C_2_-ceramide in our study. Finally, in the absence of gastric distension, superfusion of the nerve trunks with 1 UI/100 µL sphingomyelinase (Fig. 2h) revealed a significant decrease in the mean amplitude of duodenal contractions (78±5% of control, paired t test, P<0.05, df = 3) as well as a 12-fold increase of ceramide (unpaired t test, P<0.01, df = 7). Taken together, all these results indicate that the conduction of excitation without action potentials requires the recurrent production of ceramide along the nerve fibres.

**Figure 3:**
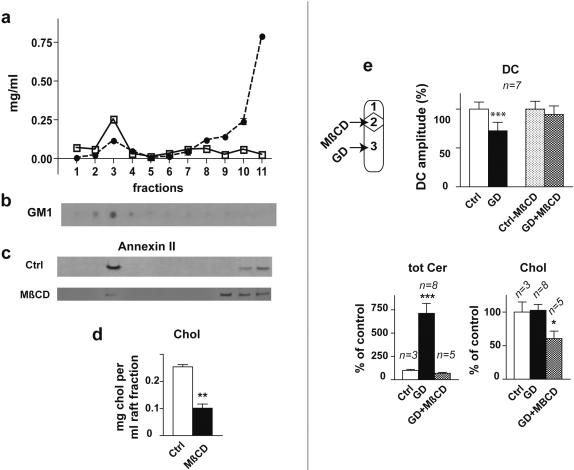
Characterization of lipid rafts isolated from the nerve trunks and their role in the organization of GIR. a, quantification of the total protein • and cholesterol □ in sucrose gradient density fractions. The low density fraction (3) exhibited high cholesterol content and low protein/cholesterol ratio compared to the high density fractions. b, c, Western blot analysis of the gradient fractions showed the enrichment of the fraction 3 in ganglioside GM1 and annexin II. c, d, decrease in annexin II (Western blot) in the rafts fraction isolated from the nerve trunks and depletion of cholesterol by 10 mM MβCD (mean of four assays from one experiment, see methods). Ctrl, control; MβCD, after treatment with MβCD. e, effects of superfusion of the nerve trunks with 10 mM MβCD for 30 min on GIR, ceramide and cholesterol content of the nerve trunks. Ctrl, control; GD, gastric distension; Ctrl-MβCD, control in the presence of MβCD; GD+MβCD, gastric distension in the presence of MβCD. Differences with control were significant in a Student's t test, *** p<0.001; ** p<0.01; * p<0.05 or non significant (ns).

Interestingly, some membrane proteins and sphingolipids are organized in sphingolipid/cholesterol-rich microdomains [18]–[20]. One of these types of domains, also called lipid rafts, may be involved in the conduction of excitation without action potentials along the nerve fibres. We first determined the presence of lipid rafts in the nerve trunks connecting the coeliac plexus to the viscera. Detergent resistant membranes (DRMs) were successfully isolated from the nerve trunk membranes treated with 0.5% Triton X-100 at 4°C. After sucrose gradient fractionation, the light density membrane fraction was obtained and was significantly enriched in cholesterol and ganglioside GM1 as expected for the lipid raft characterization [18], [21], (Figure 3a, b). Furthermore, a different protein pattern was determined by SDS-PAGE between the low and the high density fractions (Figure S2). A proteomic approach by MALDI-TOF/MS investigations of the prominent bands from the low density fractions led to the identification of two known lipid raft proteins annexin II [22] and tubulin [23]. The details of the m/z peptides profile of these lipid raft markers are given in Figure S3. As shown in Figure 3c, further analysis using the monoclonal antibody against annexin II confirmed that the lipid raft fraction 3 was enriched in annexin II. After 10 mM methyl-β-cyclodextrin (MβCD) treatment which disrupts the lipid rafts by cholesterol depletion [24]–[26], cholesterol content in the lipid raft fraction decreased significantly (40±3% from control, P<0.01, df = 10, Fig. 3d). In addition, the immunoblot of annexin II showed a decrease in the lipid raft fraction 3 and a shift of annexin II from the low to the high density fractions (Fig. 3c). Taken together these results strongly support the presence of lipid rafts in the nerve fibres organizing the GIR. We then checked whether the lipid rafts disruption would affect the GIR. When the nerve trunks were superfused with 10 mM MβCD, the mean amplitude of duodenal contractions following gastric distension was 93±11% of control (paired t test, non significant, df = 6) which revealed an inhibition of the GIR (Fig. 3e). We also found that 10 mM MβCD treatment of the nerve trunks prevented the increase in ceramide during GIR (unpaired t test, non significant, df = 6) while decreasing significantly their cholesterol content (60.6±11% from control, unpaired t test, P<0.05, df = 6, Fig. 3e). All these results support the idea that the integrity of lipid rafts is critical for the neuronal conduction of excitation without action potentials. Ceramide being a hydrophobic second messenger is located within the neuronal membrane [27]. So it is likely that other second messengers located in the cytoplasm are activated in cascade to ensure the recurrent production of ceramide leading to the conduction of excitation.

### Involvement of the intracellular calcium stores and of NO-cGMP pathway

**Figure 4:**
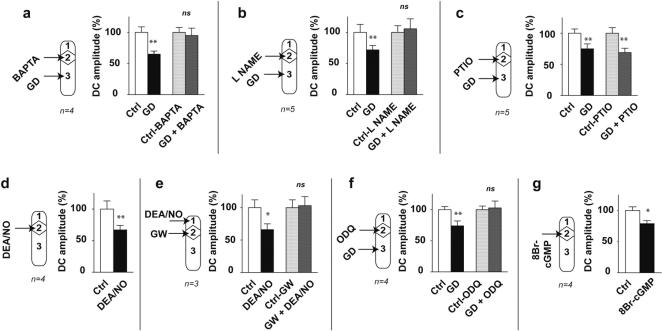
Calcium, NO and cGMP are activated in cascade within the nerve fibres during the organization of the GIR. The GIR is blocked by superfusion of the nerve trunks with 13 µM BAPTA A/M (a), 1 mM L-NAME (b), 2 µM ODQ (f) and is unaffected by 3 µM carboxy-PTIO (c). Inhibition of duodenal contractions mimicking the GIR is triggered by superfusion of the nerve trunks with 40 µM DEA/NO (d) or 200 µM 8Br-cGMP (g). Inhibition of duodenal contractions triggered by superfusion of the ganglion compartment with 40 µM DEA/NO is blocked by superfusion of the nerve trunks with 16 µM GW4869 for 30 min (e). Differences with control were significant in a Student's t test, *** p<0.001; ** p<0.01; * p<0.05 or non significant (ns).

We have previously demonstrated that superfusion of the nerve trunks with cadmium chloride or with low calcium solution did not affect the GIR, which ruled out the involvement of extracellular calcium in the conduction of excitation [1]. To determine whether ceramide could activate the release of intracellular calcium, we analysed the effects of BAPTA/AM, a permeant calcium chelator [28]–[30]. When the nerve trunks connecting the coeliac plexus to the viscera were selectively superfused with 13 µM BAPTA/AM for at least 30 min, gastric distension failed to affect significantly the duodenal contractions which revealed an inhibition of the GIR (paired t test, non significant, df = 3, Fig. 4a). This leads to the conclusion that intracellular calcium release is required for the neuronal conduction of excitation without action potentials.

The neuronal nitric oxide synthase (NOS) being calcium dependent [31], the increase in intracellular calcium concentration could have led to nitric oxide production. To check this hypothesis we analysed the effects of drugs interfering with the NO–cGMP (nitric oxide- guanosine 3′, 5′-cyclic monophosphate) pathway. When the nerve trunks were selectively superfused with 1 mM N^ω^-nitro-L-arginine methyl ester (L-NAME), a permeant inhibitor of the NO synthase, for at least 30 min, gastric distension did not significantly affect the duodenal contractions which revealed an inhibition of the GIR (paired t test, non significant, df = 4, Fig. 4b). This indicates that the activation of the NO synthase and then the production of NO are required for the neuronal conduction of excitation without action potentials. To determine the specificity of the conduction of excitation, we hypothesized that the NO production remained essentially located within the intracellular compartment. So we predicted that the use of 2-(4-carboxyphenyl)-4,4,5,5-tetramethylimidazoline-1-oxyl-3-oxide (Carboxy PTIO), a NO scavenger of too great a size to penetrate the intracellular compartment from the superfusing solution, would not affect the GIR. Indeed, when the nerve trunks were selectively superfused with 3 µM Carboxy PTIO for at least 20 min, following gastric distension the mean amplitude of duodenal contractions was 69±7% of control which revealed a significant inhibition (paired t test, P<0.01, df = 4, Fig. 4c). So Carboxy PTIO was without effect on the GIR which indicates that NO produced within the nerve fibres during the conduction of excitation without action potentials does not diffuse sufficiently through the neuronal membranes to activate other fibres. This explains why the conduction of excitation remains limited to the specific network activated by gastric distension. In a previous work we have shown that superfusion of the coeliac plexus with carboxyPTIO inhibited the GIR [2]. This indicated that NO is the neurotransmitter released in the extracellular space by the gastric afferent fibres to activate the ganglionic neurons. Taken together, these results and those of the present study show that a same molecule, NO, is involved in the conduction of excitation along the autonomic nerve fibres and in neuronal communication within the prevertebral ganglia.

Interestingly, in the absence of gastric distension, superfusion of the nerve trunks with 40 µM diethylamine/nitric oxide complex sodium (DEA/NO, a NO donor) significantly decreased the mean amplitude of duodenal contractions (67±7% of control, paired t test, P<0.01, df = 3, Fig. 4d). This phenomenon occurred with a mean latency of 7±1 min, lasted 17±4 min and mimicked the GIR. In the absence of gastric distension, superfusion of the coeliac plexus with 40 µM DEA/NO also significantly decreased the mean amplitude of duodenal contractions (66±9% of control, paired t test, P<0.05, df = 2, Fig. 4e). This inhibition was blocked by superfusion of the nerve trunks with 16 µM GW 4869 (paired t test, non significant, df = 2, Fig. 4e). All these results confirm that production of NO within the nerve fibres is involved in the conduction of excitation without action potentials and in ceramide production.

When the nerve trunks were selectively superfused with 2 µM 1H-[1], [2], [4]oxadiazolo[4,3-a]quinoxalin-1-one (ODQ, a selective inhibitor of the NO-activated soluble guanylate cyclase) for at least 30 min, the gastric distension failed to affect the duodenal contractions significantly (paired t test, non significant, df = 3, Fig. 4f). This suggested that the activation of the NO-cGMP pathway is required for the neuronal conduction of excitation without action potentials.

Finally, in the absence of gastric distension, superfusion of the nerve trunks with 200 µM 8-bromo-guanosine 3′, 5′-cyclic monophosphate (8-Br-cGMP, a permeant analogue of cGMP) significantly decreased the mean amplitude of duodenal contractions (79±6% of control, paired t test, P<0.05, df = 3, Fig. 4g). This process occurred with a mean latency of 8±2 min, lasted 23±5 min and mimicked the GIR. This result shows that an increase in cGMP within the nerve fibres can trigger a conduction of excitation without action potentials leading to an inhibition of the duodenal motility. This result supports the involvement of cGMP in the conduction of excitation without action potentials.

### Activation by ceramide of the Ca^++^-NO-cGMP pathway

Once the involvement of the Ca^++^-NO-cGMP pathway during the neuronal conduction of excitation without action potentials was established, it remained to demonstrate that this pathway was activated by the ceramide produced within the nerve fibres. To check this hypothesis, we tried to trigger a neuronal conduction of excitation by superfusing the coeliac plexus with C_2−_ceramide while the release of intracellular calcium or the NO synthase or guanylate cyclase activity downstream was blocked by superfusion of the nerve trunks with BAPTA/AM, L-NAME or ODQ respectively.

**Figure 5:**
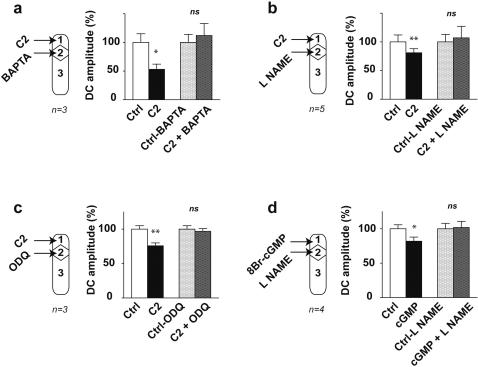
Activation by ceramide of the Ca^++^-NO-cGMP pathway. Inhibition of duodenal contractions triggered by superfusion of the ganglion with 6 µM C2-ceramide was blocked after superfusion of the nerve trunks with 13 µM BAPTA/AM (a), 1 mM L-NAME (b) or 2 µM ODQ (c). Inhibition of duodenal contractions triggered by superfusion of the ganglion with 200 µM 8Br-cGMP was blocked after superfusion of the nerve trunks with 1mM L-NAME (d). Differences with control were significant in a Student's t test, ** p<0.01; * p<0.05 or non significant (ns).

In the absence of gastric distension, selective superfusion of the nerve trunks with 13 µM BAPTA/AM for at least 30 min abolished the inhibition of duodenal contractions triggered by superfusion of the coeliac plexus with 6 µM C_2−_ceramide (paired t test, non significant, df = 2, Fig. 5a). This result indicates that the neuronal conduction of excitation without action potentials triggered by C_2−_ceramide is inhibited when the release of intracellular calcium downstream is blocked.

In the absence of gastric distension selective superfusion the nerve trunks with 1 mM L-NAME for at least 30 min, abolished the inhibition of duodenal contractions triggered by superfusion of the coeliac plexus with 6 µM C_2−_ceramide (paired t test, non significant, df = 4, Fig. 5b). This result indicates that the neuronal conduction of excitation without action potentials triggered by C_2−_ceramide is inhibited when the NO synthase activity downstream is blocked.

Finally, in the absence of gastric distension, selective superfusion the nerve trunks with 2 µM ODQ for at least 30 min abolished the inhibition of duodenal contractions triggered by superfusion of the coeliac plexus with 6 µM C_2−_ceramide, (paired t test, non significant, df = 2, Fig. 5c). This result indicates that the neuronal conduction of excitation without action potentials triggered by C_2_-ceramide is inhibited if the activity of the guanylate cyclase downstream is blocked.

All these results lead to the conclusion that during the neuronal conduction of excitation without action potentials endogenous ceramide activates the Ca^++^-NO-cGMP pathway.

### Recurrent activation of the NO-cGMP pathway

To conduct the excitation without action potentials, the activation of the NO-cGMP pathway could occur in cascade along the whole length of the nerve fibres. To check this hypothesis we tried to trigger a neuronal conduction of excitation by superfusion of the coeliac plexus with 8-Br-cGMP while the NO synthase activity downstream was blocked by superfusing the nerve trunks with L-NAME.

In the absence of gastric distension, selective superfusion of the nerve trunks with 1 mM L-NAME for at least 30 min abolished the inhibition of duodenal contractions triggered by superfusion of the coeliac plexus with 200 µM 8-Br-cGMP (paired t test, non significant, df = 3, Fig. 5d). So the excitation without action potentials triggered by 8-Br-cGMP failed to propagate when NO synthase activity downstream was blocked. However, it has never been demonstrated that cGMP could directly activate NO synthase. Therefore the most likely explanation for our result is that 8-Br-cGMP had activated downstream a cascade of other sequences including ceramide then NO production and cGMP synthesis. These sequences had been interrupted at the NO synthesis level which blocked the cascade and prevented the conductance of excitation.

## Discussion

Our study based on an integrated physiological model proposes a new neuronal mechanism: the conduction of excitation without action potentials. From the mean latency of the reflex (4 min) and the length of the afferent and efferent fibres connecting the stomach to the duodenum via the coeliac plexus (4 cm), we estimated that this conduction occurs at a mean speed of 1 cm per minute [1], [2]. This is intermediate between the fastest axonal flow described (400 mm a day) [32] and the lowest speed of propagation for action potentials (0.1 m per second for unmyelinated fibres). The excitation propagates over considerable distances along the nerve fibres (several centimetres) which is fundamentally different from all the previously described mechanisms of neuronal communication without action potentials. These mechanisms are based on slow electrotonic variations in the membrane potential of non-spiking neurones propagating only over very short distances [33]–[36].

**Figure 6:**
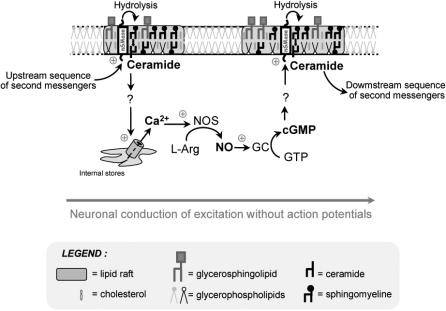
Model of a neuronal conduction of excitation without action potentials. Activation of neutral sphingomyelinase triggers ceramide production in rafts then the release of calcium from intracellular stores which activates the NO-cGMP pathway. This pathway activates downstream sphingomyelinase in neighbouring rafts which ensures the propagation of the excitation. L-Arg: L-arginine, NOS: NO synthase, GC: guanylate cyclase, nSmase: neutral sphingomyelinase.

The mechanism we propose requires the recurrent production of ceramide, probably from rafts to rafts along the fibres. This is achieved through the activation in cascade of the same second messenger sequence involving calcium, NO and GMPc (Fig. 6). This adds a new role for ceramide which is already known to be involved in major biological processes such as cell growth, differentiation, apoptosis and senescence [37]–[39] as well as modulation of neuronal synaptic activation [40], [41]. The mechanosensor triggering the neuronal conduction of excitation still remains to be determined. It could be sphingomyelinase itself since it has been demonstrated in vascular endothelial cells that neutral sphingomyelinase was activated by a transient mechanical stimulus [42], [43].

We have demonstrated previously that during the GIR, NO is the neurotransmitter released by the gastric afferent fibres to activate the ganglionic neurons [2]. So during the functioning of the autonomic networks involved in the organization of the GIR, the same molecule, NO, is involved in both the conduction of excitation without action potentials within the nerve fibres and in the communication between the neurons of the ganglionic networks. This is fundamentally different from the classic functioning of the neuronal networks which involves a conduction of excitation based on the propagation of action potentials (due to ionic fluxes) and the activation of membrane receptors due to the release of neurotransmitters. Thus, the properties of the mechanism we propose open up new prospects concerning the functioning of neuronal networks.

## Materials and Methods

All procedures regarding the handling of experimental animals have been approved by the French Ministry of Agriculture and are in agreement with the European Communities Council Directive (86/609/EEC).

### *In vitro* experimentation

We have used an *in vitro* integrated physiological model. Experiments were performed on 174 rabbits as already published [2]. The organ bath has been modified and now has three adjacent compartments (Figure S1): the first contained the coeliac plexus and the proximal part of the peripheral nerve fibres connecting the coeliac plexus to the viscera, the second contained the main part of these fibres and the third the distal part of these fibres and the viscera (stomach and duodenum). A myotomy was performed in the pyloric region to interrupt the enteric nervous pathways between the stomach and duodenum. All these compartments could be superfused independently with drugs, their separation being achieved with Vaseline grease. The technical characteristics of this set-up make it possible to trigger excitation without action potentials in the proximal part of the nerve fibres (by agonist superfusion of the coeliac plexus) and to block it downstream by selectively superfusing with drugs the compartment containing the main part of these fibres. This protocol was used in particular to identify the endogenous molecules involved in the conduction along the nerve fibres of excitation without action potentials. Gastric distensions used to trigger the GIR were within a physiological range since they bring the stomach to a volume similar to that observed when full of nutriments. Intraluminal duodenal pressure was measured with a water-filled balloon connected via a catheter to a home-made pressure transducer (M. Manneville). The duodenal contractions were stored using the PowerLab system (ADInstruments Pty Ltd, Castle Hill, Australia) with the Chart v4.1.2 software. Data were then exported in the Matlab format for processing using a home-made software (Dr P. Sanchez). The time of occurrence and the amplitude of each duodenal contraction were detected. The mean of the amplitude per minute was then calculated and used to assess the duodenal motility during the periods of time to analyse.

### Lipid analysis. Lipids were analyzed at the Lipidomic Platform of IFR30- Toulouse Génopole

The nerve trunks (6 mm length) connecting the coeliac plexus to the viscera (stomach and duodenum) were harvested 5 min following gastric distension or the different treatments, washed in Phosphate Buffer Saline and immediately frozen in liquid nitrogen. Lipids were extracted in chloroform/methanol/water (1∶2∶0.9, v∶v∶v) in a Dounce homogenizer in the presence of standards, then analysed by gas liquid chromatography for neutral lipids including cholesterol [44] or for sphingomyelin and ceramide [15] mass content. Phospholipid content was determined by total lipid phosphorus [45]. Results are expressed as the mean±SEM according to the number of experiments. Comparison with controls were analysed using a Student's unpaired t test and differences were significant at P<0.05

### Lipid rafts isolation

The nerve trunks were harvested and rinsed in cold homogenization buffer (HB) consisting of 20 mM Tris-HCl pH 7.4, 150 mM NaCl, 10 µg leupeptin, 10 µg/ml aprotinin, 1 mM benzamidine, 1 mM PMSF and 5% sucrose. Unless otherwise stated, all the experiments were performed at 4°C with the appropriate protease inhibitor concentration. For total membrane fractionation, the nerve trunks were homogenized in about 3 vol. (w/v) of HB with a Potter-Elvehjem homogenizer as previously reported [21]. The homogenate was then centrifuged at 10,000 rpm for 10 min and the pellet was adjusted to 1–1.5 mg/ml protein with HB. The resulting membrane preparation then underwent a detergent treatment with 0.5% Triton X-100 for 30 min. The extract (2 ml) containing 1.3 mg/ml protein was mixed in a 12-ml centrifuge tube with an equal volume of 80% sucrose in the same buffer, giving a suspension of 40% sucrose. A discontinuous gradient was prepared by overlaying 5 ml of 30% sucrose and then 2 ml of 5% sucrose both in 20 mM Tris HCl pH 7.4 buffer. The tubes were centrifuged at 35,000 rpm for 17 h at 4°C in a TH-641 Sorvall rotor. Fractions (11 each of 1 ml) were harvested gently from the top of the gradient. Control experiments were run as follows: without detergent, DRMs prepared with 1% Brij 98 at 37°C or 60 mM octyl glucoside at 4°C.

### Raft protein and cholesterol content determination

The protein concentration was determined by using either the BCA protein assay kit (Pierce biotechnology, Inc) with bovine serum albumin as the standard or by the Bio-Rad DC™ (detergent compatible) protein assay with immunoglobulin G as the standard. For cholesterol depletion, MβCD was applied to the nerve trunks as previously reported [46]. The cholesterol concentration in lipid raft fractions and membrane preparations was enzymatically determined by using the colorimetric method from Boehringer Mannheim following manufacturer's instructions. The results are expressed as the mean±SEM. Comparison with control was analysed using a Student's unpaired t test and difference was significant at P<0.01

### Electrophoresis and Western blotting

SDS-PAGE on 4–20% Tris-HCl precast gels (Bio-Rad) were performed under reducing conditions according to Laemmli [47]. The separated proteins were transferred to a nitrocellulose membrane and probed with the primary antibody against annexin II (from Santa Cruz Biotechnology, Germany) and HRP-conjugated secondary antibody. For lipid rafts ganglioside GM1, 2.5 µl of each sucrose gradient fraction was spotted on a nitrocellulose membrane before incubation with 1 µg cholera toxin B-HRP conjugated. Blots were developed by electrochemiluminescence (ECL) detection reagents according to manufacturer's instructions (Amersham Biosciences, France).

### Mass spectrometry analysis

Proteins in excised gel plugs were digested as described previously [21] using sequencing grade modified porcine trypsin (12.5 ng/ml, Promega, Madison, WI). The peptides were extracted, dried in a vacuum, centrifuged, and redissolved in 10 to 20 µl of 0.1% TFA (trifluoric acid). The peptide mixture resulting from protein digestion was analyzed using an Ettan pro MALDI time-of-flight mass spectrometer (Amersham biosciences, Uppsala, Sweden) in positive ion reflector mode. 0.3 µl of the peptide mixture was co-crystallized on the MALDI target with an equal amount of matrix solution (3 mg/ml of α-cyano-4-hydroxycinnamic acid in 50% acetonitrile) in the presence of 0.5% TFA. Alternatively, peptide mixtures derived from proteins were desalted and concentrated using zip tips (Millipore Bedford, MA) and deposited onto the MALDI target by elution with the matrix solution. Proteins were identified by the Profound (ProteoMetrics, LLC, New-York, NY) and the Mascot (Matrix science Ltd, London, UK) software that query comprehensive sequence databases. The presented data is representative of at least four experiments with similar results.

### Statistical analysis during *in vitro* experimentation

Under our experimental conditions, the duodenal motility was maintained in a satisfactory state for 6–7 h during which the amplitude of contractions decreased slowly. This slight diminution with time of the duodenal contractions was taken into account when comparing the successive effects of gastric distension. For this purpose, the mean amplitude of the duodenal contractions preceding each distension or agonist superfusion was taken as 100% and no more than 5 distensions or superfusions were performed on the same preparation. The effects of gastric distension or superfusions were analysed from the mean amplitude of the duodenal contractions. For statistical analysis, the mean values of this parameter before and after distension or superfusion were then compared using Student's paired t test, including variance analysis (ANOVA). Values, expressed as mean±SEM, were taken to be statistically different if P<0.05.

## Supporting Information

Figure S1 In vitro set up used to study GIR. The organ bath contains three adjacent compartments receiving the coeliac plexus, the nerve fibres and the viscera (stomach and duodenum). Each compartment is superfused independently. The duodenal motility is recorded by manometric technics.
(0.12 MB TIF)

Figure S2 SDS-PAGE protein patterns of raft and non-raft fractions from the nerve trunks. Equal total protein of each fraction (5 mg) from the low density fractions (3 and in some experiments 4), the high density fractions (9–10), the pelleted fraction (11) and total material from the starting detergent-resistant membrane (T) were separated by 4–20% SDS-PAGE and the gel was visualized by silver staining. Molecular weight standards are indicated (M).
(4.02 MB TIF)

Figure S3 Peptide mass fingerprints of Annexin II. Mass spectrograms of the indicated polypeptides bands digested by trypsin and subjected to MALDI-TOF/MS analysis as described in Materials and Methods. The molecular masses of the peptides originating from the identified protein are indicated. Ty indicates the molecular mass of the trypsin peptides.
(0.28 MB TIF)
