# Supplementary figures and images for: Neuronal Conduction of Excitation without Action Potentials Based on Ceramide Production
Source: PLoS One. 2007 Jul 18;2(7):e612. doi: 10.1371/journal.pone.0000612 (PMC1906860; doi:10.1371/journal.pone.0000612)

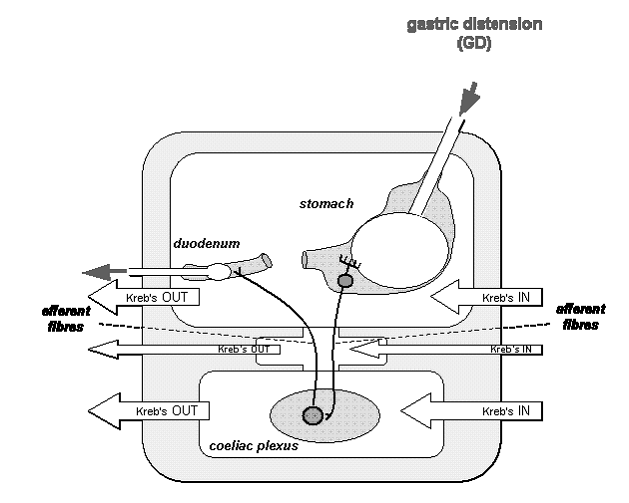

Supplement: Figure S1 — In vitro set up used to study GIR. The organ bath contains three adjacent compartments receiving the coeliac plexus, the nerve fibres and the viscera (stomach and duodenum). Each compartment is superfused independently. The duodenal motility is recorded by manometric technics. (0.12 MB TIF) [file pone.0000612.s001.tif]

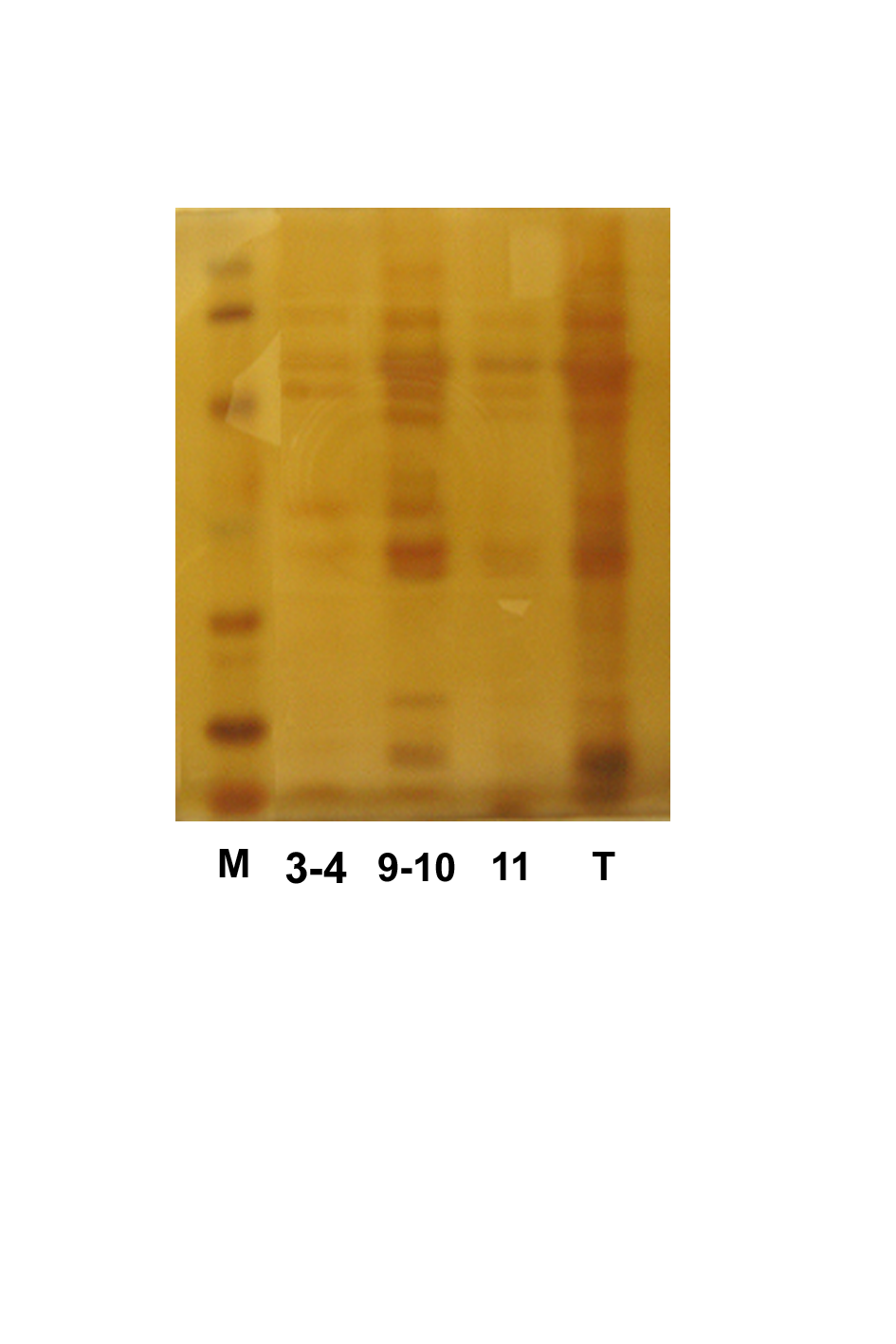

Supplement: Figure S2 — SDS-PAGE protein patterns of raft and non-raft fractions from the nerve trunks. Equal total protein of each fraction (5 mg) from the low density fractions (3 and in some experiments 4), the high density fractions (9–10), the pelleted fraction (11) and total material from the starting detergent-resistant membrane (T) were separated by 4–20% SDS-PAGE and the gel was visualized by silver staining. Molecular weight standards are indicated (M). (4.02 MB TIF) [file pone.0000612.s002.tif]

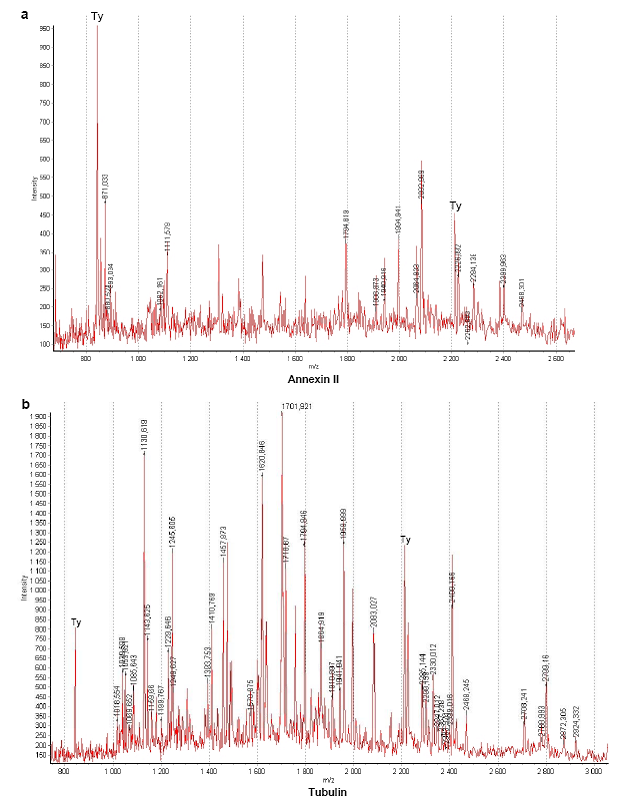

Supplement: Figure S3 — Peptide mass fingerprints of Annexin II. Mass spectrograms of the indicated polypeptides bands digested by trypsin and subjected to MALDI-TOF/MS analysis as described in Materials and Methods. The molecular masses of the peptides originating from the identified protein are indicated. Ty indicates the molecular mass of the trypsin peptides. (0.28 MB TIF) [file pone.0000612.s003.tif]
